# Supplementary material for: HIVprotI: an integrated web based platform for prediction and design of HIV proteins inhibitors
Source: J Cheminform. 2018 Mar 9;10:12. doi: 10.1186/s13321-018-0266-y (PMC5845081; doi:10.1186/s13321-018-0266-y)
Supplement: Supplementary file 2 — Additional file 2. Source code of HIVProtI web server. [file 13321_2018_266_MOESM2_ESM.zip › HIVprotI_Source-code/hivprotI/weka-3-6-6/doc/weka/core/converters/ArffLoader.ArffReader.html]

ArffLoader.ArffReader


---


|  |  |  |
| --- | --- | --- |
| **Overview** \| **Package** \| **Class** \| **Tree** \| **Deprecated** \| **Index** \| **Help** \| **Weka's home** | |  |
| **PREV CLASS**   **NEXT CLASS** | **FRAMES**    **NO FRAMES**     **All Classes** |  |
| SUMMARY: NESTED \| FIELD \| CONSTR \| METHOD | DETAIL: FIELD \| CONSTR \| METHOD |  |


---


## weka.core.converters Class ArffLoader.ArffReader

```
java.lang.Object
  weka.core.converters.ArffLoader.ArffReader
```

**All Implemented Interfaces:**: RevisionHandler

**Enclosing class:**: ArffLoader

---

``` public static class ArffLoader.ArffReader extends java.lang.Object implements RevisionHandler ```

Reads data from an ARFF file, either in incremental or batch mode.

Typical code for batch usage:

```
 BufferedReader reader = new BufferedReader(new FileReader("/some/where/file.arff"));
 ArffReader arff = new ArffReader(reader);
 Instances data = arff.getData();
 data.setClassIndex(data.numAttributes() - 1);
```

Typical code for incremental usage:

```
 BufferedReader reader = new BufferedReader(new FileReader("/some/where/file.arff"));
 ArffReader arff = new ArffReader(reader, 1000);
 Instances data = arff.getStructure();
 data.setClassIndex(data.numAttributes() - 1);
 Instance inst;
 while ((inst = arff.readInstance(data)) != null) {
   data.add(inst);
 }
```

**Version:**
:   $Revision: 7703 $

**Author:**
:   Eibe Frank (eibe@cs.waikato.ac.nz), Len Trigg (trigg@cs.waikato.ac.nz), fracpete (fracpete at waikato dot ac dot nz)

---

**Constructor Summary**

`ArffLoader.ArffReader(java.io.Reader reader)`
  
          Reads the data completely from the reader.

`ArffLoader.ArffReader(java.io.Reader reader,
Instances template,
int lines)`
  
          Reads the data without header according to the specified template.

`ArffLoader.ArffReader(java.io.Reader reader,
Instances template,
int lines,
int capacity)`
  
          Initializes the reader without reading the header according to the
specified template.

`ArffLoader.ArffReader(java.io.Reader reader,
int capacity)`
  
          Reads only the header and reserves the specified space for instances.


| **Method Summary** | |
| --- | --- |
| `Instances` | `getData()`             Returns the data that was read |
| `int` | `getLineNo()`             returns the current line number |
| `java.lang.String` | `getRevision()`             Returns the revision string. |
| `Instances` | `getStructure()`             Returns the header format |
| `Instance` | `readInstance(Instances structure)`             Reads a single instance using the tokenizer and returns it. |
| `Instance` | `readInstance(Instances structure, boolean flag)`             Reads a single instance using the tokenizer and returns it. |

**Methods inherited from class java.lang.Object**

`equals, getClass, hashCode, notify, notifyAll, toString, wait, wait, wait`

**Constructor Detail**

### ArffLoader.ArffReader

```
public ArffLoader.ArffReader(java.io.Reader reader)
                      throws java.io.IOException
```

:   Reads the data completely from the reader. The data can be accessed
    via the `getData()` method.

    **Parameters:**: `reader` - the reader to use **Throws:**: `java.io.IOException` - if something goes wrong **See Also:**: `getData()`

---


### ArffLoader.ArffReader

```
public ArffLoader.ArffReader(java.io.Reader reader,
                             int capacity)
                      throws java.io.IOException
```

:   Reads only the header and reserves the specified space for instances.
    Further instances can be read via `readInstance()`.

    **Parameters:**: `reader` - the reader to use: `capacity` - the capacity of the new dataset **Throws:**: `java.io.IOException` - if something goes wrong: `java.lang.IllegalArgumentException` - if capacity is negative **See Also:**: `getStructure()`, `readInstance(Instances)`

---


### ArffLoader.ArffReader

```
public ArffLoader.ArffReader(java.io.Reader reader,
                             Instances template,
                             int lines)
                      throws java.io.IOException
```

:   Reads the data without header according to the specified template.
    The data can be accessed via the `getData()` method.

    **Parameters:**: `reader` - the reader to use: `template` - the template header: `lines` - the lines read so far **Throws:**: `java.io.IOException` - if something goes wrong **See Also:**: `getData()`

---


### ArffLoader.ArffReader

```
public ArffLoader.ArffReader(java.io.Reader reader,
                             Instances template,
                             int lines,
                             int capacity)
                      throws java.io.IOException
```

:   Initializes the reader without reading the header according to the
    specified template. The data must be read via the
    `readInstance()` method.

    **Parameters:**: `reader` - the reader to use: `template` - the template header: `lines` - the lines read so far: `capacity` - the capacity of the new dataset **Throws:**: `java.io.IOException` - if something goes wrong **See Also:**: `getData()`


**Method Detail**

### getLineNo

```
public int getLineNo()
```

:   returns the current line number

    :   **Returns:**: the current line number

---


### readInstance

```
public Instance readInstance(Instances structure)
                      throws java.io.IOException
```

:   Reads a single instance using the tokenizer and returns it.

    :   **Parameters:**: `structure` - the dataset header information, will get updated in case of string or relational attributes **Returns:**: null if end of file has been reached **Throws:**: `java.io.IOException` - if the information is not read successfully

---


### readInstance

```
public Instance readInstance(Instances structure,
                             boolean flag)
                      throws java.io.IOException
```

:   Reads a single instance using the tokenizer and returns it.

    :   **Parameters:**: `structure` - the dataset header information, will get updated in case of string or relational attributes: `flag` - if method should test for carriage return after each instance **Returns:**: null if end of file has been reached **Throws:**: `java.io.IOException` - if the information is not read successfully

---


### getStructure

```
public Instances getStructure()
```

:   Returns the header format

    :   **Returns:**: the header format

---


### getData

```
public Instances getData()
```

:   Returns the data that was read

    :   **Returns:**: the data

---


### getRevision

```
public java.lang.String getRevision()
```

:   Returns the revision string.

    :   **Specified by:**: `getRevision` in interface `RevisionHandler`
    :   **Returns:**: the revision


---


|  |  |  |
| --- | --- | --- |
| **Overview** \| **Package** \| **Class** \| **Tree** \| **Deprecated** \| **Index** \| **Help** \| **Weka's home** | |  |
| **PREV CLASS**   **NEXT CLASS** | **FRAMES**    **NO FRAMES**     **All Classes** |  |
| SUMMARY: NESTED \| FIELD \| CONSTR \| METHOD | DETAIL: FIELD \| CONSTR \| METHOD |  |


---
